# Application of virtual and mixed reality for 3D visualization in intracranial aneurysm surgery planning: a systematic review

**DOI:** 10.3389/fsurg.2023.1227510

**Published:** 2023-09-27

**Authors:** Elisa Colombo, Bart Lutters, Tessa Kos, Tristan van Doormaal

**Affiliations:** ^1^ Department of Neurosurgery and Klinisches Neurozentrum Zurich ZH, Universität Zürich; Universitätsspital Zürich, Zurich, Switzerland; ^2^ Julius Center for Health Sciences and Primary Care, Medical Humanities, University Medical Center Utrecht, Utrecht, Netherlands; ^3^ Image Science Institute, University Medical Center Utrecht, Utrecht, Netherlands

**Keywords:** cerebrovascular surgery, intracranial aneurysms, virtual reality, mixed reality, 3D visualization

## Abstract

**Background:**

Precise preoperative anatomical visualization and understanding of an intracranial aneurysm (IA) are fundamental for surgical planning and increased intraoperative confidence. Application of virtual reality (VR) and mixed reality (MR), thus three-dimensional (3D) visualization of IAs could be significant in surgical planning. Authors provide an up-to-date overview of VR and MR applied to IA surgery, with specific focus on tailoring of the surgical treatment.

**Methods:**

A systematic analysis of the literature was performed in accordance with the PRISMA guidelines. Pubmed, and Embase were searched to identify studies reporting use of MR and VR 3D visualization in IA surgery during the last 25 years. Type and number of IAs, category of input scan, visualization techniques (screen, glasses or head set), inclusion of haptic feedback, tested population (residents, fellows, attending neurosurgeons), and aim of the study (surgical planning/rehearsal, neurosurgical training, methodological validation) were noted.

**Results:**

Twenty-eight studies were included. Eighteen studies (64.3%) applied VR, and 10 (35.7%) used MR. A positive impact on surgical planning was documented by 19 studies (67.9%): 17 studies (60.7%) chose the tailoring of the surgical approach as primary outcome of the analysis. A more precise anatomical visualization and understanding with VR and MR was endorsed by all included studies (100%).

**Conclusion:**

Application of VR and MR to perioperative 3D visualization of IAs allowed an improved understanding of the patient-specific anatomy and surgical preparation. This review describes a tendency to utilize mostly VR-platforms, with the primary goals of a more accurate anatomical understanding, surgical planning and rehearsal.

## Introduction

Intracranial aneurysms (IAs) are pathological dilatations of cerebral arteries. IAs are relatively commonly acquired lesions occurring with a frequency ranging between 0.5% and 3% in the general population, and accounting for about 80%–85% of non-traumatic subarachnoid hemorrhages (1). Upon detection of an IA, tailoring of the optimal treatment strategy is based on careful consideration of the patient history and specific aneurysm characteristics. Treatment approaches are surgical and/or endovascular. With advances in endovascular approaches, the indications for surgical clipping of IAs have been decreased. Currently, open IA clipping is generally reserved for complex aneurysms. Successful and safe surgery of these cases depends on accurate surgical planning, which implies precise pre-operative characterization of lesion-specific anatomical features. The current gold standard imaging modality for the preoperative study of IAs is digital subtraction angiography (DSA). DSA allows a comprehensive anatomical examination of the most relevant IAs' features (i.e.,: relation to the parent vessels, neck's width, dome's regularity and orientation) at the cost of invasiveness. The role of magnetic resonance flow (MR-flow) has indeed been increasing for the diagnosis and the preoperative analysis of IAs. Nonetheless, MR studies are mostly black-and-white and visualized on two-dimensional (2D) screens. When compared to two-dimensional images, three-dimensional (3D) anatomical visualization with virtual reality (VR) and mixed reality (MR) offers a more comprehensive anatomical visualization and understanding in the perioperative phase. In a VR environment, the user is fully immersed in a simulated world. To create an immersive environment, each eye is provided with a separate image by the displays in the VR device. The user's physical movement is registered by cameras in the VR device and matched to the digital world. An MR device enhances the user's physical environment with a digital overlay, a so-called hologram. MR provides the opportunity to interact with the digital objects in the physical world through (depth) cameras and a motion sensor in the device that map out the user's surroundings and track their movements (2).

Both VR and MR techniques are increasingly adopted in neurosurgical preparation to provide a safe environment to plan surgical procedures, rehearse and foresee possible technical difficulties, and make the intraoperative phase more efficient (3).

Despite their substantial promise, a systematic analysis of the literature examining the role of MR and VR applications and their benefits as perioperative adjuncts in open IA surgery has been lacking. Authors present a comprehensive review on the topic, with the primary goal to study the true measurable benefits of using 3D visualization with MR and VR in preparation of IA surgery. This analysis thereby provides an overview of the technology used, its drawbacks and the potential future improvements.

## Materials and methods

A systematic review was performed using the Preferred Reporting Items for Systematic Reviews and Meta-Analyses (PRISMA) guidelines (4). Two reviewers (EC and TK) screened records independently, and disagreements at any stage were resolved by discussion and consensus. Two additional records were identified through reference search. The critical appraisal of the included studies was performed by means of a risk of bias score using a modified version of the Cochrane Risk of Bias tool as shown in Table 1 (5).

**Table 1:** Risk of bias score.

Author	A	B	C	D
Fellner et al. (6)	+	-	**+**	NA
Koyama et al. (7)	+	+	**+**	NA
Wong et al. (8)	+	+	**+**	NA
Bu et al. (9)	+	+	NA	NA
Mo et al. (8)	+	+	-	NA
Mori et al. (10)	+	+	-	-
Agarwal et al. (11)	+	+	**+**	NA
Nakabayashi et al. (12)	+	+	NA	NA
Bambakidis et al. (13)	+	+	**+**	NA
Di Somma et al. (14)	+	+	NA	NA
Cabrilo et al. (15)	+	+	NA	NA
Alaraj et al. (16)	+	-	**+**	NA
Kockro et al. (17)	+	+	**+**	-
Chugh et al. (18)	+	-	**+**	NA
Shono et al. (19)	+	+	NA	NA
Tucker et al. (20)	+	+	NA	NA
Eftekhar et al. (21)	+	-	-	NA
Gmeiner et al. (22)	+	+	**+**	NA
Toyooka et al. (23)	+	+	-	-
Neyazi et al. (24)	+	+	**+**	NA
Zawy Alsofy et al. (25)	+	+	**+**	NA
Haridas et al. (26)	+	+	NA	NA
Deib et al. (27)	+	-	NA	NA
Allgaier et al. (28)	+	+	**+**	NA
Li et al. (29)	+	+	NA	NA
Perin et al. (30)	+	+	**+**	-
Steineke et al. (31)	+	+	**+**	NA
Stifano et al. (32)	+	+	NA	NA

A: Appropriate eligibility criteria; B: Exposure/outcome measurement; C: Failure to adequately control confounding; D: Incomplete follow-up; NA, not applicable.

### Search strategy

The PubMed and EMBASE databases were searched to identify eligible papers. The query was performed using the Boolean operators “AND” or “OR”, and database-related filters to maximize the chance to identify articles focusing on 3D visualization through MR and VR system applied to IA surgery. The following string was entered:

((“neurosurg*"[Title/Abstract] OR “Neurosurgery"[MeSH Terms] OR “Neurosurgical Procedures"[MeSH Terms] OR “ventriculostom*"[Title/Abstract] OR “lobectom*"[Title/Abstract] OR “craniotom*"[Title/Abstract] OR “neuro surg*"[Title/Abstract] OR “neurologic surg*"[Title/Abstract]) AND (“augmented realit*"[Title/Abstract] OR “Augmented Reality"[MeSH Terms] OR “mixed realit*"[Title/Abstract] OR “virtual realit*"[Title/Abstract] OR “extended realit*"[Title/Abstract] OR “hologra*"[Title/Abstract] OR “Holography"[MeSH Terms] OR “head mounted display*"[Title/Abstract] OR “head up display*"[Title/Abstract] OR “head worn display*"[Title/Abstract] OR “Smart Glasses"[MeSH Terms])).

The most recent search was performed on November 28th 2022.

### Selection criteria

Articles were included if the following criteria were met: (1) Studies published after 1997; (2) Studies analyzing specifically the role of MR and VR in IA pre-surgical and intraoperative phases; (3) A specified 2D or 3D visualization technique as a mean to study angioarchitecture; (4) English, Italian, French or German language.

### Data extraction

The following information was extracted from all included publications: (1) study group and year of publication; (2) type and number of IA included in the analysis; (3) imaging data source (computed tomography angiography (CTA), magnetic resonance angiography (MRA), digital subtraction angiography (DSA)); (4) category of visualization techniques (screen, glasses, head-mounted device (HMD); (5) inclusion of haptic feedback; (6) aim of the study (surgical planning/rehearsal, neurosurgical training, methodological validation); (7) study population (residents, fellows, attending neurosurgeons).

### Statistical analysis

The descriptive statistical analyses were performed using R Studio. Data were presented as numbers and percentages.

## Results

**Figure 1:**
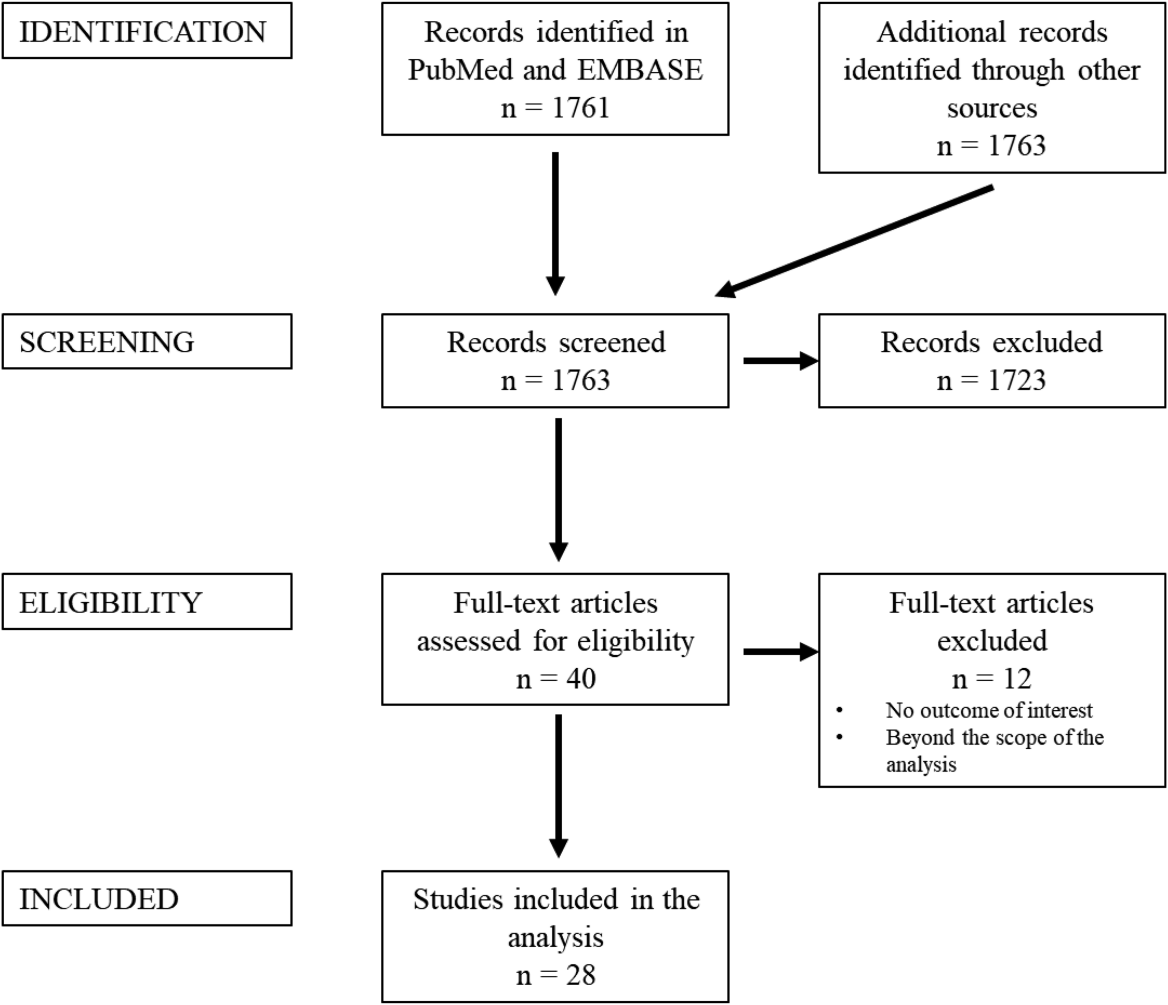
Summary of search strategy (PRISMA flow chart) for relevant studies.

A PRISMA flowchart is displayed in Figure 1. A total of 1,763 publications were screened, 40 full-text articles were assessed for eligibility and 28 studies were included in this review. Studies were excluded when considered beyond the scope for the aims of the present analysis, and/or when their outcomes were not of interest. An overview of the included studies highlighting their major goals and advantages/disadvantages of augmented reality application as perceived by the authors of the publications is illustrated respectively in Table 2 and Table 3. In Table 3, where no data was specified, it means that the authors of the publication did not express it. Table 1 provides a visual summary of the quality review of the included studies.

**Table 2:** Studies’ major goals, imaging elaboration techniques and display types, and source imaging.

Author	Year	AR	Major goals	Technology (+/-) processing tools	Display type	Source imaging
Fellner F et al.	1998	MR	Intraoperative visualization	Virtual cisternoscopy and Voxel View software	Screen	CT + MRI
Koyama T et al.	2000	MR	Surgical planning	New application program and Visual C++	Screen	NA
Wong et al.	2007	VR	• Surgical planning • Training	Dextroscope system (Bracco Diagnostics Inc.)	• Screen • Glasses/Head set	CT
Bu B et al.	2009	VR	Surgical planning	Dextroscope system (Bracco Diagnostics Inc.)	• Screen • Glasses/Head set	CT + MRI
Mo D et al.	2010	VR	Surgical planning	Dextroscope system (Bracco Diagnostics Inc.)	• Screen • Glasses/Head set	CT
Mori et al	2011	VR	Surgical planning	Mimics software	Screen	CT
Agarwal N et al	2012	VR	Training	Dextroscope system (Bracco Diagnostics Inc.)	• Screen • Glasses/Head set	CT + MRI
Nakabayashi H et al.	2012	MR	Surgical planning	AW VolumeShare, Stereo Movie Maker and QuickTime Virtual Reality	• Screen • Glasses/Head set	CT
Bambakidis NC et al.	2013	VR	Training	Selman Surgical Rehearsal Platform (Surgical Theater)	• Screen • Glasses/Head set	CT
Di Somma A et al.	2014	VR	Method validation	Dextroscope system (Bracco Diagnostics Inc.)	• Screen • Glasses/Head set	CT
Cabrilo I et al.	2014	MR	Intraoperative visualization	iPlan Workstation, Brainlab	Screen	CT + MRI + DSA
Alaraj A et al.	2015	VR	Training	Immersive Touch platform	• Screen • Glasses/Head set	CT
Kockro R et al.	2016	VR	Surgical planning	Dextroscope system (Bracco Diagnostics Inc.)	• Screen • Glasses/Head set	CT + MRI
Chung AJ et al.	2017	VR	Clipping	Selman Surgical Rehearsal Platform (surgical Theater)	• Screen • Glasses/Head set	CT
Shono N et al.	2017	MR	Surgical planning	Unity game engine, Avizo, Maya, and Leap Motion	• Screen • Glasses/Head set	CT + MRI + DSA
Tucker et al.	2017	VR	Training	Surgical Theatre	• Screen • Glasses/Head set	CT + MRI
Eftekhar B et al.	2017	MR	Intraoperative visualization	Sketchfab.com and Virtual Reality Modeling Language	Screen	DSA
Gmeiner M et al.	2018	VR	Training	New simulator and RISC Software—MEDVIS 3D	Screen	CT + DSA
Toyooka et al.	2018	MR	• Intraoperative visualization • Surgical planning	Head-up Display system and iPlan Workstation, Brainlab	Glasses/Head set	CT + MRI
Neyazi et al.	2019	VR	• Surgical planning • Training	Unity game engine and Virtual Reality Toolkit and MeVisLab	Glasses/Head set	MRI
Alsofy SZ et al.	2020	MR	Surgical planning	VR workstation connected to HTC Vive goggles and the SteamVR system and 3D Slicer	• Screen • Glasses/Head set	CT
Haridas A et al.	2020	VR	Surgical planning	Surgical Theatre	• Screen • Glasses/Head set	CT
Deib G et al.	2020	MR	Intraoperative visualization	Magic Leap One device	• Screen • Glasses/Head set	CT
Allgaier M et al.	2021	VR	Surgical planning	Unity game engine and XR Interaction Toolkit	Glasses/Head set	MRI
Li Z et al.	2021	VR	Surgical planning	Visualization Tool Kit and Unity3D platform	Glasses/Head set	CT
Perin A et al.	2021	VR	• Surgical planning • Training	Surgical Theatre	• Screen • Glasses/Head set	CT
Steineke TC et al.	2021	VR	Surgical planning	Surgical Theatre	• Screen • Glasses/Head set	CT + MRI
Stifano V et al.	2021	MR	Surgical planning	New MR application, Unity 3D, 3D Slicer and Blender	Screen Glasses/Head set	CT

AR, augmented reality; MR, mixed reality; VR, virtual reality; CT, computer tomography; MRI, magnetic resonance imaging; DSA, digital subtraction angiography; NA, not applicable.

**Table 3:** Advantages and disadvantages.

Author	Year	AR	Advantages	Disadvantages
Fellner F et al.	1998	MR	• Depth, perspective, lighting, color • Correct therapeutic decision	• Difficult visualization of perforators Intraoperative application • Operator-dependence • Time consuming
Koyama T et al.	2000	MR	Virtual manipulation	Imperfect reproduction of reality
Wong et al.	2007	VR	• Overview of the vasculature from any perspective • Haptic feedback • Training and education	• Small vessels running horizontally tend to be underestimated • Intraaneurysmal features not displayed • Time consuming
Bu B et al.	2009	VR	• Surgical rehearsal • Preoperative risk assessment	NA
Mo D et al.	2010	VR	Quick simulation	• Intraaneurysmal features not displayed
Mori et al.	2011	VR	More precise minimally invasive craniotomy planning	NA
Agarwal N et al.	2012	VR	Nonthreatening learning environment with immediate feedback	• Intraaneurysmal hemodynamics not displayed • No intrinsic information of the vessel's wall • No information about surrounding structures
Nakabayashi H et al.	2012	MR	• More effective realistic surgical simulation • Design of minimally invasive procedures	NA
Bambakidis NC et al.	2013	VR	Improved training experience, rehearsal and safety	No data on patients’ outcomes
Di Somma A et al.	2014	VR	Improved anatomical understanding	NA
Cabrilo I et al.	2014	MR	• Optimization of patient positioning and operative trajectory • Better anatomical understanding • Supportive to intraoperative orientation	• Too small cohort to objectively evaluate the real impact of Mixed Reality
Alaraj A et al.	2015	VR	Improved anatomical understanding, intuitive training experience, haptic feedback	• Intraaneurysmal hemodynamics not displayed • No intrinsic information of the vessel's wall
Kockro R et al.	2016	VR	• Intraoperative deja-vu': enhancement of surgical confidence • Stereoscopic display and manipulation • Steep learning curve • Depth perception	Retrospective analysis lacking control groups
Chung AJ et al.	2017	VR	Statistically significant improvement in time per clip used	No patient outcome nor safety of surgical clipping
Shono N et al.	2017	MR	• Optimization of intraoperative trajectory and clip placement • Archive of cases that could be used for training • Incorporation of sense, touch and hearing	• Force feedback not incorporated • Feasibility not validated • Quality of the model dependent on quality of source imaging • Cumbersome workflow
Tucker et al.	2017	VR	• Better appreciation of the surgical anatomy • Nonthreatening environment for surgical simulation and training	The application of VR for neurosurgical training should be further and better implemented
Eftekhar B et al.	2017	MR	Improved anatomical orientation	Privacy concerns
Gmeiner M et al.	2018	VR	Improved anatomical understanding, realistic experience, improved training, satisfactory haptic feedback	Not realistic for calcified aneurysms, small perforators and wall irregularities like mini-blebs
Toyooka et al.	2018	MR	Understanding of anatomy, geometry and approach	Poorer image quality of the HUD
Neyazi et al.	2019	VR	Benefit on surgical trajectory and education	No patient-specific data
Alsofy SZ et al.	2020	MR	Benefit on aneurysm detection, anatomical understanding, surgical approach, and clipping planning	• No display of small branches and perforators, nor adhesions • Great dependence on the quality of input data
Haridas A et al.	2020	VR	• Detailed evaluation of the patient-specific anatomy prior to surgery • Better understanding of the complex anatomy in high resolution	NA
Deib G et al.	2020	MR	• Better preoperative anatomical understanding	NA
Allgaier M et al.	2021	VR	• Steep learning curve after adequate training • Improved anatomical understanding and planning of the surgical approach	Difficult first-time use. No real patients data
Li Z et al.	2021	VR	• Better anatomical understanding • Training environment • Enhanced surgical confidence	• Small number of experiments • Experimental equipment is relatively backward, resulting in inaccurate results
Perin A et al.	2021	VR	Improved anatomical understanding, realistic experience, improved training	• No haptic feedback: no simulation of dissection and tissue handling • Small study sample • Costs: limited diffusion
Steineke TC et al.	2021	VR	• Benefits on preoperative planning and rehearsal with decreased intraoperative times • Improved training experience and increased intraoperative efficiency	• Lack of an agreed on and validated complexity scoring system • Small sample size and not a varied population of surgeons tested
Stifano V et al.	2021	MR	• Better anatomical understanding • Optimization and customization of surgical planning • Valuable training tool	• Low comfort and maneuverability • Dependence of the model on the quality of the source imaging • Limited number of patients and users

AR, augmented reality; MR, mixed reality; VR, virtual reality; NA, not applicable.

### Virtual reality

Virtual reality implies the use of a system which generates a complete immersion in a digital environment, that could provide a realistic simulation of the surgical approach (16). This type of technology was applied by 18 of the studies (64.3%) (8–11, 13, 14, 16–18, 20, 22, 24, 26, 28–31, 33), with a total of 321 aneurysms included in the studies. The VR systems that were mostly used were the Dextroscope system (Bracco Diagnostics Inc., Milan, Italy), documented by 6 studies (33%) (8, 9, 11, 14, 17, 33), and Surgical Theater (Surgical Theater Inc., Los Angeles, CA), utilized by 4 studies (22%) (20, 26, 30, 31). Thirteen studies (72%) chose the combination of screen and glasses/HMD as preferred visualization method (8, 9, 11, 13, 14, 16–18, 20, 26, 30, 31, 33). Exclusive use of a 2D visualization of the CT images represented the most relevant imaging source in 10 studies (56%) (8, 10, 13, 14, 16, 18, 26, 29, 30, 33).

• Preoperative planning:

None of the studies of this subgroup applied VR intraoperatively. 16 of the studies (89%) focused on the pre-operative planning of the surgical approach. The benefit of VR application for preoperative planning was qualitatively assessed using Likert scales and the Think Aloud Method, specifically for evaluation of anatomical understanding, depth perception and visualization of the surgical trajectory perceived by nine study groups (8, 14, 16, 17, 22, 24, 28, 30, 31).

• Benefit on training:

Eight studies (44%) assessed the impact of this technology on training neurosurgical residents, focusing on the benefits of VR with regard to realistic anatomical understanding, haptic feedback satisfaction and enhancement of surgical confidence (8, 11, 13, 16, 20, 24, 30). User satisfaction was assessed by means of Likert Scales and the Think-Aloud Method.

• Impact on patients:

Three studies (17%) evaluated the potential effect of 3D visualization in VR on clinical outcomes (10, 17, 30). None of the 18 studies aimed to evaluate the impact of surgical planning with VR on patient safety, and only one study (5.6%) aimed to assess the benefits of VR on patient education and understanding of the surgical procedure (8).

• Perceived disadvantages:

The lack of information on intra-aneurysmal hemodynamics and vessel wall characteristics was also reported as a disadvantage (8, 11, 16, 22). Furthermore, small vessels and perforating arteries tended to be underestimated or not displayed (8, 22).

### Mixed reality

Conceptually, MR differs from VR in that it integrates a virtual environment with the real world, whereas the latter is a full immersion in a virtual environment. MR provides an interaction with digital objects in the real world. Ten of the collected studies applied this technology (6, 7, 12, 15, 19, 21, 23, 25, 27, 32), with a total of 183 analyzed IAs. In this subgroup, there was indeed no homogeneity among the MR systems used: each group utilized a center-specific system and different softwares for the segmentations and the post-processing of the images. 3D visualization occurred by means of a combination of screen and glasses/HMD in 6 out of 10 studies (60%) (7, 12, 19, 25, 27, 32). The remaining 4 studies (40%) utilized solely screen for image visualization (6, 7, 15, 21). CT as exclusive imaging input source was used by 3 studies (30%) (12, 25, 27), and 4 studies (40%) chose a multimodal imaging source (6, 15, 19, 23). Only one group (10%) used DSA only as the input source (21). None of the studies in this subgroup utilized MRI as the exclusive imaging source.

• Preoperative planning:

Four studies (40%) used this technology for both the surgical planning and intra-operative guidance (6, 15, 21, 27). The major goal documented in this subgroup was again planning of the best surgical approach, as documented by 5 studies (50%). Similarly to the VR-subgroup, the advantages perceived for the preoperative planning were based on an improved anatomical orientation, better depth perception and more adequate understanding of the surgical approach (6, 21, 25). The major outcomes of these studies were mostly evaluated through Likert Scales for a qualitative assessment. Only 2 studies (20%) performed a structured statistical analysis to examine the outcomes (23, 25).

• Benefit on training:

One of the studies in this subgroup aimed to assess the impact of MR visualization on neurosurgical training, testing the technology on residents neurosurgeons (32).

• Impact on patients:

None of the studies in this subgroup aimed to validate the impact of MR visualization on patient education/safety or clinical outcomes.

• Perceived disadvantages:

The most relevant drawback reported in the MR-subgroup was the difficult, if not impossible, visualization of small vessels and perforators, and the dependence of the segmentation on the quality of the input data (5/10 studies, 50%) (6, 7, 21, 23, 25).

## Discussion

The present analysis represents an up-to-date systematic review of all published studies, which applied perioperative 3D visualization through MR and VR to IA microsurgery from 1997 to November 2022.

A relevant aspect emerging from the present analysis is the lack of measurable hardcore values to quantitatively examine the real added value of VR and MR applied to open IA surgery. While a qualitative assessment of the benefits of these 3D technologies is possible using Likert Scales and the Think Aloud Method, the absence of objective qualitative parameters makes the analysis partial and may hinder objective comparisons among the different 3D modalities, especially when a structured statistical analysis is not performed. Under this premise, this systematic review suggests an improved anatomical understanding, a better depth perception and a nonthreatening learning environment to be the most relevant perceived advantages of VR, MR applied for IA surgery planning, compared to conventional visualization strategies. The 3D and realistic replication of the cerebrovascular anatomy could help the acquisition of procedural motor skills, and enhance surgical orientation and confidence (34).

Most of the included studies used multimodal imaging input to create a more informative 3D vascular model, overcoming the disadvantages of exclusive use of one imaging modality. While CTA allows for a precise understanding of aneurysmal size and shape, provides detailed information on the parent vessel, and anatomical relationships with the skull base, combination with digital subtraction angiography (DSA) or MR-flow adds information on the flow patterns (22, 35). Nonetheless, none of the studies in the present cohort integrated hemodynamic information to the 3D visualization. Furthermore, the combination of CT and MR imaging provides important information on vessel/aneurysm spatial relationships with the parenchyma and the cisternal system, which allow a better surgical orientation (11).

As far as visualization techniques are concerned, merging glasses or HMD's with 2D visualization of 3D vascular models enhances the perception of spatial position and surgical orientation (8). Glasses and HDMs may also allow a more intuitive and immersive interaction with the 3D models (36).

The use of VR. MR and RV does not come without limitations. The studies published so far are mostly retrospective, with small sample sizes and no control groups. The analyzed studies examine almost exclusively aneurysms treated in elective settings, with specific focus on anterior circulation IAs, and rarely provide information on patient functional outcomes. The lack of an objective strategy to qualitatively assess the benefits of these technologies represents a major bias as well. While Likert-scales or Think-Aloud Method are mostly applied to evaluate the intuitiveness and the satisfaction of the users, no standardized, agreed on quantitative scores have been provided yet. To obviate this absence, objective parameters such as size of the aneurysmal dome, width of the neck, orientation of the dome, distance of the aneurysm from relevant anatomical structures should be noted, when using VR and/or MR, validated and combined into quantitative scores. The difficulty of these analyses may lie in the paucity of data and in the novelty of these technologies, which are still not available in every center. Their diffusion may also be limited by their often not affordable costs. Another relevant aspect resulting from the paucity and diversity of the available data is the lack of unified criteria to provide an objective appraisal of the current literature. The advantages and the disadvantages reported for each paper come mostly from the appraisal and experience of the original authors. With further implementation of these technologies and gathering of more extensive and unified data, this limitation could be obviated. Furthermore, segmentation of intracranial vessels and fine anatomical structures is still highly dependent on the quality of input data, which makes the integration of hemodynamic information, small vessels or intramural particularities difficult. The integration of hemodynamic information into a 3D preoperative study of IAs may help characterize their angioarchitecture more accurately. This information may provide major advantages on the tailoring of their treatment in a pathology-specific way. Such a tailoring could potentially increase intraoperative safety and therapeutic efficiency.

## Conclusion

This analysis endorses the promising role of MR and VR to provide a more accurate aneurysm-specific anatomical visualization and understanding. The absence of a standardized set of quantitative parameters to provide an objective assessment of the real benefit of these technologies on training in IA surgery should be a major drive for future studies on the topic.

Furthermore, integration of hemodynamic analysis to the 3D visualization may also be a promising avenue for future research.

## Data Availability

The original contributions presented in the study are included in the article/Supplementary Material, further inquiries can be directed to the corresponding author.
